# Role of ILC2s in Solid Tumors: Facilitate or Inhibit?

**DOI:** 10.3389/fimmu.2022.886045

**Published:** 2022-06-03

**Authors:** Lige Wu, Weiqing Zhao, Shuxian Tang, Rui Chen, Mei Ji, Xin Yang

**Affiliations:** Department of Oncology, Third Affiliated Hospital of Soochow University, Changzhou, China

**Keywords:** group 2 innate lymphoid cell, tumor, inflammation, immunity, cytokine

## Abstract

Group 2 innate lymphoid cells (ILC2s) are important mediators of type 2 immunity and play an important role in allergic diseases, helminth infections, and tissue fibrosis. However, the role of ILC2s in tumor immunity requires further elucidation. Studies over the past decade have reported that ILC2s play a promoting or suppressing role in different tumors. Here we reviewed the role of ILC2s in solid tumors demonstrating that ILC2s act as a crucial regulator in tumor immunity. We proposed that ILC2s could be an important predictor for tumor prognosis and a new therapeutic target after immunotherapy resistance. In conclusion, our study shed new light on modifying and targeting ILC2s for anti-tumor immunotherapy.

## Introduction

Innate lymphoid cells (ILCs) are the counterparts of T cells in innate immunity. According to the development and function, ILCs are divided into five subsets—NK cells, ILC1s, ILC2s, ILC3s, and lymphoid tissue-inducer cells (LTi cells). Human ILC2s were discovered in the fetal gut in 2011. Mjosberg et al. initially defined this cell population as Lineage negative CRTH2^+^ CD127^+^ CD161^+^ cells; these cells expressed considerable amounts of IL-13 after *in vitro* stimulation with IL-25 and IL-33 (1). ILC2s have since been found in myriad healthy and diseased human tissues, such as lung, nasal polyps, kidney, intestine, skin, and tumors (2, 3). Recent discovery of CRTH2^−^ ILC2s suggests that CRTH2 is no longer suitable as a common marker for all human ILC2s (hILC2s) (4).

## ILC2 Differentiation and Migration

Although ILCs are morphologically similar to lymphocytes and can produce high levels of T-helper cytokines, ILCs lack antigen receptor and lineage (Lin) markers and can expand and function normally in T-cell-deficient nude mice (5, 6). Therefore, ILC and T-cell differentiation and development are traditionally believed to be independent of each other (7). However, recent studies have shown that ILCs are a branch of neonatal T-cell progenitors that colonize peripheral tissues in the third trimester (8, 9). The CD4^−^ CD8^−^ double-negative (DN) T cells may differentiate into ILCs at DN1/ETP and DN2-DN3 transition stage, and this change is influenced by the intensity of Notch signaling and by E-ID protein and Bcl11b activity (10). In addition, Shin et al. reported in particular that the differentiation of tissue-resident ILC2s may be because of DN2 ineffectively rearranging their γ/δ loci (10). The exact source of ILC2s that colonize in peripheral tissues and organs remains unknown. In mouse lung tissue, IL-18R^−^ ST2^+^ ILC2s are differentiated *in situ* from immature IL-18R1^+^ ST2^−^ innate lymphoid cell precursors (ILCPs) (11). Many studies have reported that tissue-resident human ILCs (hILCs) expand and maintain activity through self-renewal during inflammation and homeostasis (12–14). It can be inferred that initially colonized hILCs possess partial stem cell properties. Although human ILCPs have been detected in adult and neonatal lungs by single-cell RNAseq of RORα tracer (15), the origin of ILC2s in human tissue needs to be confirmed by further studies.

In mice, ILC2s are divided into tissue-resident natural ILC2s (nILC2s) and circulating inflammatory ILC2s (iILC2s). In mice, pulmonary nILC2s are defined as Lin^−^ ST2^+^ KLRG1^int^ cells which respond to IL-33 and pulmonary iILC2s are defined as Lin^−^ ST2^−^ KLRG1^hi^ cells which respond to IL-25 (16). However, the murine small intestinal lamina propria iILC2s are defined as Lin^−^ CD25^−^ ST2^−^ cells (17). In a parabiotic mice model of inhibiting commensal bacteria with antibiotics, resting ILC2s residing in intestinal lamina propria are activated by IL-33 or helminths into iILC2s, which migrate to various extraintestinal tissues through sphingosine 1-phosphate (S1P)-mediated chemotaxis to participate in anti-helminth defense and tissue repair (18). hILC2s were previously considered to be exclusively tissue-resident, but this couldn’t explain the increased abundance of ILC2s and other ILC subsets in peripheral blood of American cutaneous leishmaniasis patients compared with healthy volunteers (19). The latest research shows that, CD45RO^+^ ILC2s are derived from resting CD45RA^+^ ILC2s in airway inflammation and are regarded as human equivalent of the iILC2 subset (20). However, whether human intestinal ILC2s can be activated into iILC2s and migrate requires further investigation. Collectively, the pool of peripheral ILC2s formed in the early stage of innate immunity may consist of both tissue-resident ILC2s and migratory iILC2s.

## ILC2 Function and Regulation

ILC2s are known to mirror the function of CD4^+^ Th2 and are crucial mediator of type 2 immunity. IL-25 and IL-33 can strongly activate human and murine ILC2s *via* NF-κB and MAPK pathways. Activated ILC2s secrete a variety of cytokines to regulate effector cell function. For instance, murine ILC2-derived IL-4 stimulates Th2 differentiation and B cells to secrete IgG1 and IgE (21–23); murine ILC2-derived IL-5 facilitates eosinophil accumulation and eotaxin production (24); murine ILC2-derived IL-9 drives the recruitment of mast cells and enhances the secretion of IL-5 and IL-13 through the autocrine loop (25, 26); human ILC2-derived IL-10 reduces Th responses and maintains epithelial integrity in regulating grass-pollen allergy (27); human and murine ILC2-derived IL-13 induces mucus secretion in epithelial cells, smooth muscle cell spasm, and tissue fibrosis (28–30); In mouse bone marrow, ILC2-derived GM-CSF promotes the recovery of HSPCs from 5-FU-induced stress (31). In addition to secreting cytokines, ILC2s can modulate T cell function through direct interactions. In mouse lung, ILC2s directly activate T cell through Ag presentation by MHCII, and blocking MHCII interactions completely prevents the ability of ILC2s to stimulate DO11.10 T cell proliferation (32). This MHCII-mediated CD4^+^ T cell activation was also observed in *Nippostrongylus brasiliensis*-infected mouse models, and co-stimulatory molecule CD80/86 was also detected in part of ILC2s (33). Activated T cells produce IL-2 to promote murine ILC2 proliferation and function, which in turn facilitate parasitic helminth expulsion (33). ILC2s in mice are kind of antigen-presenting cells and its expression of MHCII appeared to be enhanced by STAT6 signaling (34). Human and murine ILC2s express both ICOS and ICOS-L (35). In mice, ICOS-L on ILC2s can bind with ICOS on the same or separate ILC2s in cis or trans formation to promote ILC2 function and homeostasis; alternatively, ICOS-L on ILC2s can interact with ICOS on Tregs, which leads to Treg accumulation and cytokine production (36). Murine ILC2s also express the co-stimulatory molecule OX40L, which interacts with OX40 on Th2 to maintain Th2-mediated type 2 immunity and interacts with OX40 on Tregs to promote Treg survival and proliferation (37).

In addition to activating cytokines (IL25 and IL-33), ILC2s are regulated by a variety of co-stimulatory and suppressive cytokines. In some cases, co-stimulatory cytokines help activating cytokines optimally activate ILC2s by upregulating the expression of GATA3; co-stimulatory cytokines include γc family cytokines and TNF superfamily (38). In mice, γc family cytokines (IL-2, IL-4, IL-7, IL-9, and TSLP) upregulate GATA3 *via* JAK/STAT pathways; while TNF superfamily (TNFSF15 and TNFSF18) acts *via* NF-κB and MAPK pathways (38). In both human and mice, suppressive cytokines (Type 1 IFNs, IFN-γ, and IL-27) downregulate GATA3 expression *via* IFN-stimulated gene factor 3 and STAT1, thereby inhibiting ILC2 function and activity; in human, IL-10 and TGF-β suppress the production of IL-4, IL-5, and IL-13 only when ILC2s response to IL-33 or IL-33 + TSLP (38–40). Interestingly, TGF-β1 deficiency has shown to suppress murine ILC2 proliferation and IL-13 secretion (41).

PD-1 is a metabolic and suppressive immune checkpoint for ILC2s. In a high-fat diet mice model, TNF-induced PD-L1^hi^ M1 macrophages inhibit the function of PD-1^+^ ILC2s *via* PD-1/PD-L1 interaction, leading to impaired glucose tolerance (42). In a mouse model of airway hyperreactivity, PD-1 deficiency shifts ILC2 metabolism to an anaerobic type (glycolysis, glutaminolysis and methionine catabolism) and enhances the activation and proliferation of ILC2s (43). The latest metabolomic and nutrient receptor analysis showed that hILC2s consumed amino acids to maintain high level of oxidative phosphorylation in a steady status, and the functional status of hILC2s relied on glycolysis and the mammalian target of rapamycin (44). These results suggest that activated ILC2s are low in oxygen dependence and mainly rely on glycolysis to maintain function; this metabolic profile of ILC2s makes it likely to adapt to the hypoxic tumor microenvironment (TME) and function in the TME. In addition, PD-1 negatively regulates the proliferation and function of human and murine KLRG1^+^ ILC2s through inhibiting STAT5 phosphorylation (45). In mice, *Pdcd1* knockdown significantly increases nuclear STAT5 in KLRG1^+^ ILC2s; in human, anti-PD-1 antibody along with rhIL-33 treatment can significantly increase the secretion of type 2 cytokines by KLRG1^+^ ILC2s (45). PD-1 upregulation was also detected in kinds of tumor-infiltrating ILC2s (TILC2s), which will be discussed below.

## ILC2 Plasticity and Heterogeneity

ILC2s demonstrate functional plasticity and can be converted into ILC1/ILC3-like cells in specific conditions. In human and mice, IL-12, IL-1β and IL-18 can switch the ILC2 phenotype to IFN-γ-producing ILC1-like cells by upregulating T-bet (46, 47); conversely, IL-4 can reverse this effect and maintain the ILC2 phenotype *via* GATA3 upregulation (48, 49). Recent study has classified hILC2s into more detailed subsets. According to the expression of CCR10, c-Kit (CD117) and CCR6, hILC2s was divided into 3 subgroups: CCR10^+^ ILC2s, c-Kit^+^ ILC2s and c-Kit^−^ ILC2s (50). The phenotype of c-Kit^+^ hILC2s can switch to ILC3-like cells when exposed to IL-1β and IL-23 in some pathological conditions. TGF-β in the microenvironment can upregulate the expression of IL-23R in c-Kit^−^ hILC2s in the presence of IL-1β, and the response to IL-23 can lead to c-Kit^−^ hILC2s differentiating into ILC3-like cells that produce IL-17 (51). In mice, under the regulation of Notch signaling, pulmonary nILC2s can switch to iILC2s, which produce IL-13 and IL-17; pulmonary iILC2s express high levels of GATA3 and also low amounts of RORγt, which is a key regulator for ILC3 differentiation and function (52, 53). The Notch transcriptional complex directly binds to the *Rorc* gene (encode RORγt) site and promotes its expression, so that iILC2s have the characteristics of both ILC2 and ILC3 (52). Moreover, murine iILC2s can switch to nILC2-like cells or ILC3-like cells which contribute to helminth defense and anti-fungi immunity (16).

ILC2s possess obvious heterogeneity. Mass cytometry and full-length single-cell RNAseq have shown that hILC2s exhibit distinct phenotypic and transcriptional signatures in different tissues (54, 55). For instance, hILC2s in peripheral blood and tonsil highly express *TNFSF10*, *TNFRSF19* and *CD200R1*, whereas hILC2s in lung highly express *IL1RL1* and *IL17RB* (55). CD69 on hILC2s was detected restrictively to skin, mucosa and spleen, whereas ICOS was mainly restricted to mucosal hILC2s (54). Collectively, ILC2s are highly regulated by the local tissue microenvironment and cytokine milieu.

## ILC2s in Solid Tumors

The role of ILC2s in solid tumors is currently unclear. In this review, we compared all relevant original studies and summarized some of the properties of ILC2s in solid tumors.

## Gastric Carcinoma (GC)

GC is one of the most common malignancies worldwide. *H. pylori* (Hp) infection is considered a class I carcinogen of GC, and the gastric body predominant type of chronic atrophic gastritis (CAG) caused by Hp infection often develops into GC (56). Chronic inflammation can damage gastric glands and lead to gastric metaplasia, a kind of precancerous lesion (57). Here we describe this pathogenic process as the “CAG-GC chain”. ILC2s are the major ILC subset in the murine stomach and highly express IL-33 receptor (ST2) at steady state (58). The frequency of ILC2s is increased in peripheral blood mononuclear cells (PBMCs) of patients with GC (59). Li et al. (60) measured the ratio between type 1 (IFN-γ) and type 2 (IL-4/IL-5/IL-13) cytokines in clinical blood samples, and found that type 1 immunity was impaired in patients with CAG and GC, whereas type 2 immunity was induced. This effect was more pronounced in Hp^+^ patients and was enhanced with the development of the “CAG-GC chain” caused by Hp infection. When inoculating mice with Hp *via* intragastric gavages, the researchers subsequently demonstrated that Hp infection significantly increased ILC2 and Th2 levels in gastric homogenate, accompanied by GATA3 upregulation (60). These results suggest that ILC2s play a facilitating role in the Hp-mediated “CAG-GC chain”, but the specific molecular mechanism still needs to be further studied. In mice, tuft cell-derived IL-25 stimulates ILC2s to release IL-13, a growth factor for tuft cells, and this circle drives early metaplastic remodeling and gastric tumorigenesis (61). Genetic ablation of murine ILC2s, tuft cells or antibody neutralization of ILC2-derived type 2 cytokines suppresses gastric tumor growth (61). Single-cell RNAseq of gastric leukocytes have revealed that murine ILC2s highly express the glucocorticoid and androgen receptors; glucocorticoids and androgens synergistically inhibit the transcription of ILC2-derived IL-13, and simultaneous deficiency of glucocorticoids and androgens in mice results in the development of gastric inflammation and metaplasia (62). These murine experiments suggest that ILC2 blockade could inhibit the occurrence and development of gastric cancer, and glucocorticoid or androgen treatment also has some therapeutic potential. However, it is still controversial whether ILC2s play a major role in the process of GC tumorigenesis. In a mouse model of GC, mast cells were proved to be the major effector cells of IL-33 and could promote GC by recruiting macrophages; the frequencies of ILC2s and Tregs were comparable between tumor and normal tissues regardless of ST2 deficiency in mice (63). More studies on IL-33/ST2 pathways in gastric TME need to be conducted to identify the most decisive factors.

## Colorectal Cancer (CRC)

In the past few years, IL-33 had been identified as an important cytokine in CRC tumorigenesis. A recent study has shown that IL-33 directly promotes murine CRC proliferation by upregulating COX2/PGE2 (64). In mice, epithelium-derived CRC cells generate IL-33 during polyposis and that IL-33 activates at least two cell types, subepithelial myofibroblasts and mast cells, to form a tissue microenvironment favorable to polyposis (65). sST2, a soluble form of the IL-33 receptor, can neutralize IL-33 in TME, thereby suppressing tumor growth, metastasis and tumor angiogenesis (66). Moreover, immunohistochemical analysis of tumor tissue samples from a large number of CRC patients showed that the upregulation of IL-33/ST2 was significantly correlated with an early tumor stage, but not with the prognosis of patients (67). This suggests that IL-33 and its effector cells may play a more important role in tumorigenesis, compared with tumor progression. Colonic lamina propria ILC2s also express ST2 in steady state and TME (68, 69), and play a considerable role in the construction of TME.

ILC2s are not typically found in the gut of healthy individuals, whereas they are found in the gut of patients with CRC (70). The PD-1 expression level of TILC2s varies in different stages of CRC. In human, PD1^low^ ILC2s are dominant in early CRC tumors, whereas PD1^high^ ILC2s are dominant in late CRC tumors (71). In mice, ILC2s in advanced CRC highly express *Hs3st1* (encodes the effector product that catalyzes heparan sulfate biosynthesis) and *Pdcd1* (encodes immune checkpoint PD-1); deficiency of PD1 or HS3ST1 in murine ILC2s can considerably inhibit CRC tumor proliferation (71). Peroxisome proliferator-activated receptor γ (PPARγ) had been proved to directly regulate PD-1 expression on ILC2s (72). Recent study has found that ILC2s in human and mouse CRC express PPARγ, which maintains ILC2 secretion of IL-5 and IL-13 and the pro-tumor effect of ILC2s (73). These results suggest that PD-1^+^ ILC2s may play a facilitating role in CRC. However, two recently published studies contradict these findings. One of the studies found that both human and mouse CRC tissue had considerably higher ILC2 levels than paracancerous tissue; in a mouse model, ILC2-derived IL-9 could activate CD8^+^ T cells to inhibit CRC tumor growth and using anti-CD90.2 to block ILC2s in nude mouse (lacking T cells) could promote tumor growth, whereas intravenously injecting IL-9 inhibited tumor growth (74). Another study found that ILC2s were markedly absent in RORα-deficient mice, and this loss was accompanied by an increase in tumor burden; in human, the high ILC2 gene signature in tumor was an independent predictor of better outcome in CRC patients (75). The reason why ILC2s act in opposite ways in the same cancer requires further study.

## Hepatocellular Carcinoma (HCC)

The proportion and distribution of hILC2 in HCC may predict the prognosis of patients. Recent study has found that higher ratio of ILC2 to ILC1 in PBMCs of HCC patients is associated with prolonged survival (76). However, the higher the ratio of hILC2 abundance in tumor tissue to that in paracancerous tissue, the poorer the prognosis (77). In addition, patient clinical data have shown that microvascular invasion, HBV infection, and tumor recurrence are positively correlated with the abundance of TILC2s in patients with HCC (77). These results suggest that a tumor prognostic model may be established centered on ILC2s. In both steady state and HCC, hepatic ILC2s express ST2 and can be activated by IL-33 (77, 78). hILC2s in HCC do not express KLRG1, but highly express CD69 and core residency signature when compared with that of normal blood and tissue hILC2s; in mice, KLRG1^−^ ILC2s in HCC can induce immunosuppressive neutrophils to accumulate in tumor tissues by releasing CXCL2, thereby promoting HCC progression (77). Collectively, ILC2s promote the progression of HCC, and blocking ILC2s may have a therapeutic effect. These results also indicate that the phenotype of main TILC2 subset may vary in different experiments. To better distinguish the ILC2 subgroups, we recommend that all studies about ILC2 targeting should include a detailed phenotypic identification of major ILC2 subsets that influence experimental outcomes.

## Pancreatic Ductal Adenocarcinomas (PDAC)

In murine pancreas, resting and activated TILC2s express ST2 and PD-1 (79). In murine PDAC, IL-33 activates TILC2s to secrete CCL5, which subsequently recruits CD103^+^ DCs to TME; DCs then activate CD8^+^ T cells through antigen presentation, thereby enhancing the anti-tumor immunity (79). As previously mentioned, PD-1 is a suppressive immune checkpoint on the cell surface of ILC2s and blocking PD-1 can preserve the immune activity of ILC2s. Therapeutic anti-PD-1 antibodies can not only block PD-1 on CD8^+^ T cells to maintain the anti-tumor effect of T cells, but also block PD-1 on ILC2s to indirectly enhance the anti-tumor immunity. In murine PDAC, IL-33-mediated TILC2 expansion is accompanied by enhanced intratumoral CD8^+^ T cell infiltration and PD-1 upregulation in TILC2s, and IL-33 combined with anti-PD-1 treatment can significantly increase TILC2 abundance and reduce tumor volume in both PD-1-sensitive and PD-1-resistant tumors (79). This suggests that IL-33 combined with anti-PD-1 antibodies may break immunotherapy resistance in patients with advanced cancer. However, the role of ILC2s in PDAC is also complex and paradoxical. Another study found that PDAC tumor cells releasing IL-33 depended on the intratumoral fungal mycobiome, and genetic deletion of IL-33 or anti-fungal treatment in murine PDAC could reduce Th2 and ILC2 recruitment and improve survival (80). But whether the intratumoral fungal mycobiome affects the phenotype and function of TILC2s remains unknown. Collectively, the role of IL-33/ILC2 axis in PDAC requires further study.

## Breast Cancer

Breast cancer is the most prevalent malignancy worldwide. The frequency of hILC2s is significantly increased in surgically resected breast cancer tissue samples (3). In a 4T1 breast cancer mouse model, IL-33/ST2 axis could not only reduce NK cell cytotoxicity, but also recruit immunosuppressive cells (MDSCs and Tregs) and ST2^+^ ILC2s to accumulate in tumors (81). The abundance of MDSCs and TILC2s is associated with distant metastasis of breast cancer. In murine breast cancer, adoptive transfer of pulmonary ILC2s promotes the infiltration of IL-13Ra1^+^ MDSCs in lung metastatic nodules through IL-13/IL-13Ra1; recruited MDSCs increase the number of lung metastatic nodules and reduce the survival of tumor-bearing mice by inhibiting CD4^+^ T cells and CD8^+^ T cells and inducing Treg (82). These results indicate that ILC2s are involved in the regulation of immunosuppressive TME in breast cancer. Although anti-PD-(L)1 antibodies have shown some efficacy in the treatment of triple-negative breast cancer, the role of PD-1 in breast ILC2s has not been reported.

## Lung Cancer

Lung cancer has the highest mortality and the second morbidity among the world malignancies. The abundance of ILC2s in PBMCs and tumor tissues of patients with non-small cell lung cancer (NSCLC) is significantly higher than that of healthy donors (83). Lung ILC2s stably express ST2 in both NSCLC patient and healthy people (83). And PD-1 is highly expressed in ILC2s obtained from NSCLC patients, both at the mRNA and protein levels; PD1^high^ hILC2s isolated from tumor tissue enhance the polarization of M2 macrophages (M2-TAMs) *in vitro* by secreting IL-4 and IL-13 (83). M2-TAMs are one of the predominant tumor-infiltrating immune cell population and promote tumor growth and metastasis (84). In a lung cancer mouse model, vitamin A deficiency diet can increase the abundance of ILC2s and M2 macrophages in tumor tissue and associates with higher tumor burden and lower survival (85). A small sample analysis showed that circulating ILC2s and MDSCs were up-regulated simultaneously in patients with lung cancer (86); another study found that Treg-induced immunosuppression in mice was associated with both CD8^+^ T cell depletion and ILC2 augmentation (87). Furthermore, ILC2s are associated with lung metastasis of malignant tumors. Investigation of numerous lung metastasis mouse models has shown that ILC2-induced eosinophil can locally antagonize lung NK cell function *via* restraining NK cell glucose metabolism (glucose restriction and enhanced glycolysis), and consequently promote tumor metastasis and dissemination in the airways (88). Collectively, TILC2s in lung are associated with multiple immunosuppressive cells and are crucial regulator of immunosuppressive TME; ILC2 blockade in lung cancer may break immune resistance and become a new treatment option for patients with anti-PD-1 antibody tolerance.

## Bladder and Prostate Cancers

Bladder cancer is one of the most common tumors of urinary system, and intravesical instillation with bacillus Calmette-Guérin (BCG) is the standard treatment for patients at moderate to high risk of recurrence. The antitumor effect of BCG is mediated by induction of delayed hypersensitivity in the host. In a prospective study of non-muscle-invasive bladder cancer (NMIBC) (89), the total amount of immune cells in the urine of patients receiving BCG treatment is increased (mainly neutrophils), and urine CD14^+^ cells mainly show the phenotype of monocytic myeloid-derived suppressor cells (M-MDSCs), which play a suppressive role in anti-tumor immunity (90). Even though ILC frequency is very low in patient’s urine, the proportion of ILC2s in total urine ILCs remains elevated after patients receiving BCG treatment, and this elevation correlates with IL-13 and M-MDSC levels in urine (89). Using BCG to stimulate PBMCs isolated from healthy donor (HD) *in vitro*, it was found that amplified ILC2 population could produce large amounts of IL-13 and induce the expansion of IL-13Rα1^+^ M-MDSCs, which significantly inhibited the proliferation of both CD8^+^ and CD4^+^ T cells; and anti-IL-13 antibody could partially restore T cell proliferation (89). This BCG-induced ILC2 expansion is more enhanced in MIBC patients compared to HD (89). These results suggest that the ILC2/IL-13/M-MDSCs axis is likely to be one of the pathways mediating BCG treatment failure. Enhanced ILC2s and M-MDSCs are also observed in human prostate cancer tissue samples, and this enhancement is not evident in samples from patients with benign prostatic hyperplasia (91). In a prostate cancer mouse model, the frequency of M-MDSCs in tumor was elevated and positively correlated with TILC2s in the same tumor, whereas NKT cells were reduced (91). This suggests that functional crosstalk occurs between ILC2s and M-MDSCs. ST2 expression has been detected in murine prostate ILC2s (91), but whether prostate ILC2s express PD-1 has not been reported yet. And it remains unknown whether bladder ILC2s express ST2 and PD-1. Together, studies on bladder and prostate ILC2s are still lacking.

## Melanoma

In a melanoma mouse model, IL-33-activated TILC2s recruit eosinophils by producing GM-CSF (92). RNA-seq in CRC demonstrated that eosinophils were critical for tumor rejection and displayed an IFN-dependent profile and cytotoxic machinery (93). In murine melanoma, eosinophils inhibit primary tumor by normalizing tumor vessels and enhancing CD8^+^ T cells infiltration (94), in contrast to promote pulmonary metastasis of the melanoma cell line B16-F10 by restraining NK cell glucose metabolism in the lungs as outlined above (88). These dual effects of eosinophils on melanoma may be related to local TME in different tissues. In mice, the anti-tumor effects of IL-33/ILC2/eosinophil axis can be impaired by lactic acid produced by melanoma (95). This indicates that the pH of TME may affect ILC2 function; however, it is still unclear whether lactate molecules directly regulate cell function. ILC2s in mice melanoma express high levels of ST2 and PD-1, and co-administration of IL-33 with PD-1 blockade therapy can significantly improve the antitumor effect mediated by ILC2s (92).. PD-1 blockade on murine ILC2s in melanoma lung metastases can upregulate the production of ILC2-derived TNF-α, and TNF-α induces tumor hemorrhagic necrosis (96, 97). Furthermore, a potential interaction between ILC2s and NK cells was found in the melanoma microenvironment. In a nude mouse model of melanoma, IL-33 could respectively activate ST2^+^ NK cells and ST2^+^ ILC2s, and ILC2s suppress NK cell infiltration and function *via* CD73 (98). In murine melanoma, ILC2s promote T cell infiltration but inhibit NK cell function; research on ILC2s in human melanoma is still lacking.

## Discussion

**Figure 1:**
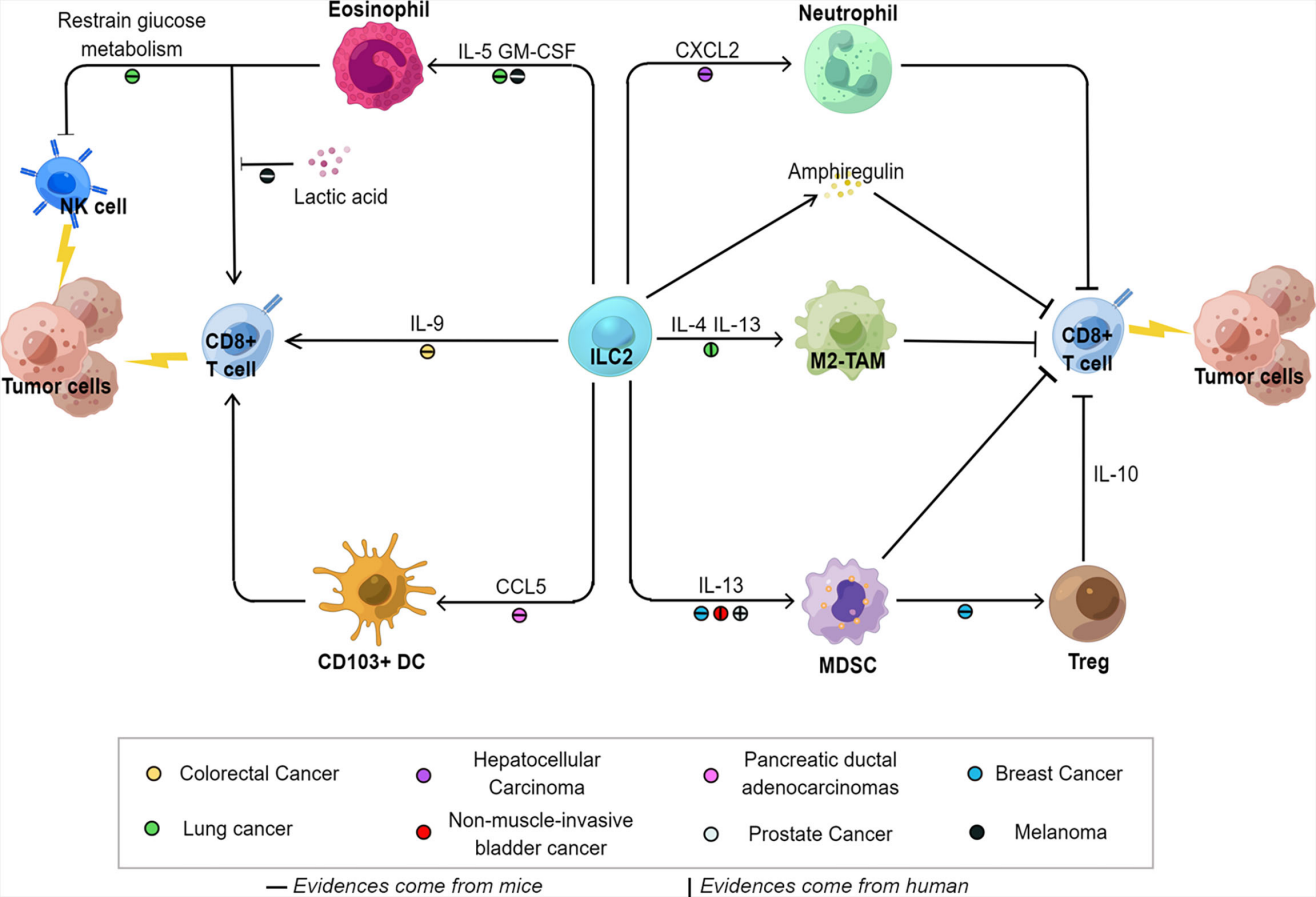
The regulatory network of ILC2s in the tumor microenvironment. Influenced by the local microenvironment, ILC2s exist the signal and functional cross-talk with other immune cells and exhibit inhibitory (left) or facilitating (right) effects on tumors. A regulatory network is formed centered on ILC2s, which act as a crucial regulator in tumor immunity. Specific tumor types associated with each pathway have been marked with colorful spots. *M2-TAM* M2 tumor-associated macrophages, *MDSC* myeloid-derived suppressor cells, *GM-CSF* granulocyte macrophage colony-stimulating factor, *CXCL2* C-X-C motif chemokine ligand 2, *CCL5* C-C motif chemokine ligand 5.

ILC2s are important mediators of type 2 immune response and are closely associated with allergic inflammation, helminth infection, and tissue fibrosis. However, the role of ILC2s in tumors remains unclear. In the present review, we examined prior studies on ILC2s in solid tumors and found that ILC2s act as a crucial regulator in tumor immunity (**Figure 1**).

## ILC2s and Cancer Prognosis

Increased abundance of ILC2s has been observed in a variety of tumors. The phenotype of ILC2s is tissue-specific, and the role of ILC2s varies in different tumors. For instance, ILC2s promote lung cancer by recruiting M2-TAMs; ILC2s recruit MDSCs to facilitate breast, bladder and prostate cancer; but ILC2s inhibit melanoma by recruiting eosinophils, which enhance the infiltration of effector T cells. Interestingly, ILC2s have been found to exert both suppressive and promoting roles in the same tumor. In CRC, ILC2s suppress tumor by producing IL-9, but maintain pro-tumor effect by expressing PPARγ. In PDAC, ILC2s recruit DCs to promote antitumor immunity, but decreasing ILC2s by genetic deletion of IL-33 improves the survival of tumor-bearing mice. In melanoma, ILC2s enhance the infiltration of effector T cells by recruiting eosinophils, but ILC2s suppress NK cell function *via* CD73. The role of ILC2s also differs in primary tumor and metastases. In melanoma, ILC2-recruited eosinophils suppress primary tumors by promoting CD8^+^ T cells but promote lung metastases by suppressing NK cells. The role of ILC2s in solid tumors is extremely complex. To better understand ILC2 function and enhance comparability between different experiments, we recommend that all studies on ILC2s should be supplemented with phenotypic identification of the main ILC2 subsets which affect experimental outcomes. In addition, type 2 immunity is enhanced while type 1 immunity is suppressed during GC tumorigenesis; the abundance and proportion of ILC2s in PBMC, tumor tissue and paracancerous tissue of HCC patients are related to the prognosis.

Current evidences suggest that ILCs are likely to be an important predictor of tumor prognosis. Here we propose a hypothesis that a tumor prognosis prediction system based on ILCs can be constructed in the following aspects: (1) the levels and proportions of type 1 and type 2 cytokines; (2) the levels and proportions of anti-tumor cytokines (mainly IL-9) and pro-tumor cytokines (IL-4, IL-13 and amphiregulin); (3) the levels and proportions of ILC and ILC2 subsets in peripheral blood and lesions. Considering the different roles of ILC2s in distinct cancer, we can construct different mathematical models based on the above aspects. Currently, the biggest limitation is that little is known about the changes in both ILC2 surface markers and ILC subset proportion during tumorigenesis and disease progression; and we still lack a general tissue-specific ILC2 test kit to overcome the detected deviation between different laboratories.

## Prospects for ILC2-Based Targeted Therapy

For precancerous lesions promoted by ILC2s (e.g. CAG-GC chain), IL-33/ST2 inhibition therapy may be used as a complement measure to surgery to lower the risk of recurrence. For tumors in which ILC2s mainly play a facilitating role, one way is to block the transcription of ILC2 cytokines or neutralize related pro-tumor cytokines; another way is to block ILC2 activation. In mice, the majority of tumor ILC2s are regulated by IL-33/ST2. For IL-33/ST2-regulated pro-tumor TILC2s, sST2 may also be a good choice in addition to traditional antibodies. For tumors in which ILC2s mainly play an inhibitory role, IL-33-combined treatment may enhance the therapeutic effect of the standard regimen.

Recently, anti-PD-1/PD-L1 antibodies have become a standard treatment for many tumors, but most patients still fail to benefit from it or relapse after treatment. Understanding the mechanism of immune resistance is the current need for clinical application. In CRC, PDAC, LC and melanoma, TILC2s have shown high PD-1 expression and may amplify the anti-PD-1 efficacy. The ILC2-MDSC axis has been identified in breast, bladder and prostate cancers, respectively. The ILC2-M2-TAM axis has been identified in lung cancer. For tumors in which ILC2s recruit immunosuppressive cells, the application of anti-PD-1 antibodies may accelerate the formation of ILC2-mediated immunosuppressive TME. These results suggest that ILC2s may predict the efficacy of PD-1 blockade therapy and may become a new therapeutic target after immunotherapy resistance. For PD-1^hi^ anti-tumor TILC2s, IL-33 combined with anti-PD-1 treatment may break through the dilemma of immunotherapy resistance in current clinical practice.

## Conclusion

In conclusion, we reviewed the original studies of ILC2s in solid tumors and found that ILC2s act as a crucial regulator in tumor immunity. Current evidences suggest that ILC2s are tissue-specific and play different roles in various tumors. It is our understanding that all studies on ILC2s should be supplemented with phenotypic identification of the main ILC2 subsets in the experiment. We also proposed the potential application value of ILC2s for tumor prognosis and analyzed the prospects of ILC2-based targeted therapies.

## Author Contributions

LW wrote the original draft and drew the illustration. MJ and XY contributed to the conceptualization. All authors participated in the revision of the manuscript. All authors contributed to the article and approved the submitted version.

## Funding

This work was supported by the National Natural Science Foundation of China (82072561); the National Natural Science Youth Foundation of China (81501971); a project funded by China Postdoctoral Science Foundation (2018M630603); the Natural Science Youth Foundation of Jiangsu Province (BK20150252); the Human Resource Summit Grant of Jiangsu Province (WSW-142); and the Youth Medical Professionals Foundation of Jiangsu Province (QNRC2016279).

## Conflict of Interest

The authors declare that the research was conducted in the absence of any commercial or financial relationships that could be construed as a potential conflict of interest.

## Publisher’s Note

All claims expressed in this article are solely those of the authors and do not necessarily represent those of their affiliated organizations, or those of the publisher, the editors and the reviewers. Any product that may be evaluated in this article, or claim that may be made by its manufacturer, is not guaranteed or endorsed by the publisher.
